# Hsa_circ_0030586 promotes epithelial–mesenchymal transition in prostate cancer via PI3K-AKT signaling

**DOI:** 10.1080/21655979.2021.2008217

**Published:** 2021-12-02

**Authors:** Guang-Cheng Luo, Lin Chen, Jiang Fang, Zhi-Jian Yan

**Affiliations:** a Department of Urology, Zhongshan Hospital Xiamen University, Xiamen, Fujian, China; b The Third Clinical Medical College, Fujian Medical University, Xiamen, Fujian, China

**Keywords:** Circular rna, has_circ_0030586, prostate cancer, pi3k-akt, epithelial–mesenchymal transition, rna sequencing

## Abstract

Circular RNAs (CircRNAs) gain importance as regulatory molecules in prostate cancer (PCa), but molecular mechanism of most circRNAs in pathogenesis of PCa remains to be studied. This study aimed to explore the role of hsa_circ_0030586 in PCa. Gene Expression Omnibus database (GSE77661) was used to screen out candidate circRNAs. Quantitative real-time PCR was used to verify the relative expressions of circRNAs, miRNAs, and genes in PCa cells. A CCK-8 assay was used to evaluate the cells’ proliferation. Transwell and wound healing assay were used to determine the cells’ migration and invasion. Western blotting and immunohistochemistry were used to detect the protein expression of PI3K/AKT signaling proteins and epithelial–mesenchymal transition (EMT) markers. Furthermore, a nude mice tumorigenesis experiment in vivo was conducted to determine the function of hsa_circ_0030586 on PCa. Our results showed that hsa_circ_0030586 is significantly upregulated in PCa cells (p < 0.05). Its circular structure was confirmed via agarose gel electrophoresis and Sanger sequencing. Interfering with hsa_circ_0030586 in PC3 cells inhibited cell proliferation, migration, and invasion and led to the significant upregulation of E-cadherin and the significant downregulation of p-AKT/AKT, IKKα, PIK3CB, and Twist (all p < 0.05). Conversely, the hsa_circ_003058 interference fragment combined with the transfection of a miR-145-3p inhibitor could reverse the above effects. In vivo tumorigenesis of the xenograft model confirmed that interfering with hsa_circ_0030586 suppressed tumor cell proliferation and inhibited PI3K-AKT signaling and EMT in PC3 cells. Hsa_circ_0030586 is significantly upregulated in PCa cells and may promote EMT via PI3K-AKT signaling.

## Introduction

Prostate cancer (PCa) is one of the most common malignant tumors in males worldwide, especially the elderly. Meanwhile, its incidence and mortality rates are substantially high [1]. About 1.6 million men are diagnosed with PCa each year [2]. Age and heredity are the main risk factors associated with PCa development [3]. It is a heterogeneous disease and can be indolent or aggressive [2]. Although most patients are diagnosed with localized PCa and could be cured via surgery and radiotherapy, up to 30% of men will relapse within 5–10 years, and a rapid biochemical relapse will lead to specific deaths in 10% of PCa patients [4]. The main PCa treatments are prostatectomy for primary PCa and androgen deprivation therapy for metastatic PCa. However, treatment options are limited for castration-resistant PCa (CRPC). Hence, it is important to discern who needs treatment to avoid unnecessary procedures that may lead to serious complications and affect the patients’ quality of life [5]. Thus, an in-depth understanding of PCa’s pathogenesis and potential therapeutic targets is urgently required.

Circular RNAs (circRNAs) are noncoding RNAs with covalently closed loop structures. They play an important role in different biological progress by acting as micro-RNA (miRNA/miR) sponges [6–8]. Recently, circRNAs have become an important regulatory molecule in PCa. For instance, the knockdown of circ_CCNB2 was shown to promote PCa radiosensitivity through autophagy repression via the miR-30b-5p/kinesin family member 18A axis [9]. Downregulated hsa_circ_0000735 reduced tumor growth in PCa via sponging miR-7 and boosted PCa’s sensitivity to docetaxel [10]. The circular RNA_LARP4 suppressed migration and invasion by upregulating forkhead box O3 alpha in PCa [11]. However, the molecular mechanisms of most circRNAs in the pathogenesis, invasion, and metastasis of PCa remain unknown and need to be further studied [12–14].

miRNAs are noncoding RNAs that are 20–25 nucleotides long and can regulate gene expression at the transcriptional or posttranscriptional level by binding to the 3′ untranslated regions [15] of mRNA. miRNAs act as regulators in various physiological processes, including proliferation, tumorigenesis, and apoptosis. A previous study has shown that miR-145 could inhibit the proliferation and invasion of LNCaP cells and was involved in the regulation of lncRNAPCGEM1 [16]. It has also been shown to affect circRNA expression in LNCaP cells [17]. A recent study showed that circCOL1A1 promotes the progression of gastric cancer cells by sponging miR-145 [18]. However, the role of miR-145-3p in PCa and its relationship with hsa_circ_003058 remain to be studied.

In this study, we intended to explore the biological role and molecular mechanism of hsa_circ_0030586 in PCa progression, which may provide new targets for the diagnosis and treatment of PCa.

## Materials and Methods

### Cell culture and transfection

Human PCa cell lines (RWPE-1, DU145, PC3, 22Rv1, LNCaP, PC-3 M-2B4, and PC-3 M-IE8) were purchased from the CellCook Company (Guangzhou, China). Cells were cultured in F-12 K medium supplemented with 10% fetal bovine serum (FBS), 100 units/mL of penicillin, and 100 μg/mL of streptomycin (Sigma-Aldrich), in 5% CO_2_ at 37°C. The circ_0030586 siRNA (5′-ATAGAAATCCAATAGGCATCA-3′) and siRNA negative control (siRNA NC: 5′-TGATGCCTATTGGATTTCTAT-3′) were synthesized by Shanghai GenePharma Co., Ltd. Additionally, the lentiviral of shRNA and shRNA negative control (shRNA-NC) was also purchased from GenePharma.

### RNA sequencing and bioinformatics analysis

The total RNA of cells was isolated using Trizol reagent (Invitrogen, USA) following the manufacturer’s instructions. The quantity and quality of total RNA were measured using an Agilent 2100 Bioanalyzer (Agilent Technologies, Palo Alto, CA, USA). The sequencing library was constructed according to the protocol of the NEB Next Ultra™ RNA Library prep kit (Illumina, San Diego, USA). Next, the differentially expressed mRNAs were analyzed and screened using R software according to their log2 ratio (siRNA/NC), absolute value of ≥1, and FDR value of ≤0.001.

Gene ontology (GO) and Kyoto Encyclopedia of Genes and Genomes (KEGG) pathway analyses were conducted by using the Database for Annotation, Visualization, and Integrated Discovery (http://david.abcc.ncifcrf.gov). The circRNA–miRNA–mRNA network was constructed and visualized using Cytoscape software.

### Quantitative real-time PCR and real-time PCR

Total RNA was extracted using Trizol reagent (Invitrogen, USA) and reverse transcribed into complementary DNA following the manufacturer’s instructions in the PrimeScript RT reagent kit (TaKaRa, Dalian, China). Then, TB GreenPremix Ex Taq II (TaKaRa, Dalian, China) was used for real-time PCR on an ABI 7500 system (Applied Biosystems, Foster City, CA, USA). Relative RNA levels were calculated using the standard 2^−ΔΔCt^ method, and glyceraldehyde-3-phosphate dehydrogenase (GAPDH) or U6 was used as an internal standard. Additionally, gel electrophoresis and Sanger sequencing were conducted to identify the cyclization site. Table 1 lists the primer sequences.

**Table 1.** Primers used in the study

Primer	Sequence (5ʹto3ʹ)	length (bp)
hsa_circ_0004365-F	AACGCTGCTGATGGGAGATAC	128
hsa_circ_0004365-R	TAACAGACACCGTGTTCACG	
hsa_circ_0004826-F	TGTCCAGAAACAGCAAACGG	137
hsa_circ_0004826-R	GAATCTGGGTGCGATTATCTGC	
hsa_circ_0005334-F	GGAACAGACATTGCAGTTCTGG	132
hsa_circ_0005334-R	TGACTGAGGTGATTGAGCAGAG	
hsa_circ_0006871-F	ACGGGTAAACCTTGCAAGAG	164
hsa_circ_0006871-R	AACAATTCGCCAGGTCTGAC	
hsa_circ_0007099-F	TTTCTGCTCAAGTCCTGTCCTC	169
hsa_circ_0007099-R	GTTCGGGAGTCTCCAGCATG	
hsa_circ_0030586-F	CTGTAGCTACCGTTCTTTTTGGC	200
hsa_circ_0030586-R	AACAATTCGCCAGGTCTGAC	
hsa_circ_0069982-F	ACAGATACACCCCCAAAGAGC	174
hsa_circ_0069982-R	TGAGGTCTTCTGTTTCCAAGGG	
hsa_circ_0030586-LF	ACGGGTAAACCTTGCAAGAG	108
hsa_circ_0030586-LR	CACAGTTCGAACAAGTGTCTGC	
GAPDH-CF	TCCTCACAGTTGCCATGTAGACCC	220
GAPDH-CR	TGCGGGCTCAATTTATAGAAACCGGG	
GAPDH-LF	AAGGTGAAGGTCGGAGTCAA	108
GAPDH-LR	AATGAAGGGGTCATTGATGG	
hsa-miR-145-3p-RT	GTCGTATCCAGTGCAGGGTCCGAGGT ATTCGCACTGGATACGACAGAACA	66
hsa-miR-145-3p-F	AAGTTACGATGGATTCCTGGA	
hsa-miR-103a-2-5p-RT	GTCGTATCCAGTGCAGGGTCCGAGGT ATTCGCACTGGATACGACCAAGGC	63
hsa-miR-103a-2-5p-F	AGTGACAGCTTCTTTACAGTG	
hsa-miR-135a-5p-RT	GTCGTATCCAGTGCAGGGTCCGAGGTA TTCGCACTGGATACGACTCACAT	65
hsa-miR-135a-5p-F	AAGCGCCTTATGGCTTTTTATTC	
hsa-U6-F	CTCGCTTCGGCAGCACA	94
hsa-U6-R	AACGCTTCACGAATTTGCGT	
hsa-PIK3CB-F	CCTTTTCTTGATTGTGCCCTCTC	101
hsa-PIK3CB-R	TGCACTTCTGACCTAAGATGCC	
hsa-ANGPT2-F	ACTTCAGCTCTTGGAACACTCC	134
hsa-ANGPT2-R	GCTTGTCTTCCATAGCTAGCAC	
hsa-CHUK-F	ATGAAGGGCCATTTGCTTCC	121
hsa-CHUK-R	ACATAGTGCACTGCTTCAGC	
hsa-E-cadherin-F	GAGAAACAGGATGGCTGAAGG	291
has-E-cadherin-R	TGAGGATGGTGTAAGCGATGG	
hsa-TWIST-F	GGAAGATCATCCCCACGCTG	124
hsa-TWIST-R	GCCATCTTGGAGTCCAGCTC	
hsa-CDK2-F	AGCTATCTGTTCCAGCTGCTC	124
hsa-CDK2-R	CAAAGTCTGCTAGCTTGATGGC	
hsa-BRCA1-F	TCAGCTTGACACAGGTTTGG	127
hsa-BRCA1-R	TTTGGCACGGTTTCTGTAGC	
hsa-GAPDH-F	GAGTCAACGGATTTGGTCGT	185
hsa-GAPDH-R	GACAAGCTTCCCGTTCTCAG	

### CCK-8 assay

The proliferation ability of PC3 cells was tested using a cell counting kit-8 (CCK-8, Beyotime Biotech, Haimen, China). First, 3 × 10^3^ cells were seeded per well in a 96-well plate. Then, 10 μL of the CCK-8 solution was added to each well. After incubation for 2 h at 37°C, absorbance at 450 nm was measured using a Spectra Max 250 spectrophotometer (ELx800, BioTek, Inc., IL, USA). The experiment was repeated three times.

### Cell migration and invasion assays

Cell migration and invasion assays in the absence and presence of Matrigel were conducted using transwell chambers (Corning, NY, USA) according to the manufacturer’s instructions. PC3 cells were transfected with 200 pmol of si-circRNA or with negative controls. After 24 h, 200 μL of the cell suspension in serum-free medium was seeded into the upper chamber, and 700 μL of culture medium supplemented with 20% FBS was added to the lower compartment. After incubation for 24 h, cotton swabs were used to wipe the cells located on the upper surfaces of the chamber, and the cells located on the lower surfaces were fixed with 4% paraformaldehyde and then stained with 0.1% crystal violet for 30 min at room temperature, respectively. The stained cells were counted in six randomly selected fields under a microscope (Leica, Wetzlar, Germany).

### Wound healing assay

PC3 cells were transfected with si-circRNA or negative controls for 24 h. Then, they were evenly inoculated into a 24-well cell culture plate. PC3 cells were trypsinized and then seeded into a 24-well plate. After the cells were completely attached, the medium was changed and mitomycin C was added to a final concentration of 1 μg/mL for 1 h to inhibit cell division. Then, a 200 μL pipette tip was used to scratch the bottom of the plate vertically against the edge of the lid. After washing three times with phosphate-buffered saline (PBS) to remove the dead cells, a basal medium was added, and cells were placed in an incubator to continue culturing. The samples were imaged under a microscope and recorded after 0, 24, and 48 h. This experiment was repeated three times.

### Western blot analysis

For Western blot (WB) analysis, the total protein of PCa cells was extracted using a RIPA lysis buffer (#P0013J; Beyotime, Beijing, China) according to the protocol, and protein concentration was measured through the BCA method. A total of 30 μg of protein was loaded and separated via sodium dodecyl sulfate–polyacrylamide gel electrophoresis and transferred onto a polyvinylidene fluoride membrane. After blocking with 5% milk at room temperature for 2 h, primary antibodies targeting E-cadherin (dilution 1:5000; Proteintech, 20,874–1AP), Twist (dilution 1:1000; Proteintech, 25,465-1-AP), p-AKT (dilution 1:2000; CST, 4060s), AKT (dilution 1:2000; CST, 2920s), IKKα (dilution 1:10,000; Abcam, ab32041), PIK3CB (dilution 1:1000; Abcam, ab151549), and GAPDH (dilution 1:8000; Proteintech, 60,004-1-1 g) were incubated at 4°C overnight. HRP-conjugated horseradish peroxidase secondary antibodies were incubated at room temperature for 2 h. Finally, blots were developed with ECL substrates. Image J software (National Institutes of Health, Bethesda, MD, USA) was used for a semiquantitative analysis, and GAPDH was used as an internal control.

### Immunohistochemistry

Immunohistochemistry (IHC) was conducted to assess the expression of epithelial–mesenchymal transition (EMT) markers (E-cadherin and Twist). The samples were blocked with a 5% BSA solution for 15 min at room temperature, and primary antibodies against E-cadherin (dilution 1:100; CST, 3195) and Twist (dilution 1:100; Proteintech, 25,465-1-AP) were incubated overnight at 4°C then washed with PBS and incubated with the secondary antibodies anti-rabbit IgG-HRP (boster, SV0002) and anti-mouse IgG-HRP (boster, SV0001). The DAB immunostaining kit (Boster Biological Technology) was subsequently used for color development, and the tissue sections were visualized using a DP-72 optical microscope (Olympus, Japan).

### Construction of stable lentivirus cell line

The lentiviral expression vector was designed to constitutively downregulate hsa_circ_0030586 via the transfection of shRNA. A vector transfecting shNC was also generated to serve as a control. PC3 cells were infected with lentivirus according to the operation manual and plated on a 24-well plate for 24 h. After plating, the lentiviral suspension packaged by GemePharma Company with a green fluorescent protein reporter gene and puromycin resistance gene was added for infection according to the optimal multiplicity of infection of 100. Cells were then cultured with the infection-enhancing solution Polybrene to increase efficiency. After 48 h of infection, puromycin was added to screen for 3–5 days, and samples were collected for quantitative real-time PCR (qRT-PCR).

### Tumor xenograft models

Animal studies were approved by the Laboratory Animal Ethics Committee of Guangzhou Forevergen Biotechnology Co., Ltd. Fourteen BALB/C mice (5–6 weeks old) were purchased from the Guangdong Medical Laboratory Animal Center and maintained in a specific pathogen-free facility. They were randomly divided into the shNC group (n = 7) and the shRNA group (n = 7). A 100 μL suspension containing 2 × 10^6^ PC3-shNC or PC3-sh2 cells was injected into the back of nude mice. Tumor volume was monitored every 3 days. When the tumor size reached 800–1,000 mm^3^, the mice were sacrificed, followed by the measurement of tumor volumes and weights.

### Statistical analysis

GraphPad_Prism_8 software was used for evaluating quantitative data in this study. Differences between groups were analyzed using the Student’s t-test. P values lower than 0.05 were considered to indicate statistically significant differences.

## Results

In this study, we used the Gene Expression Omnibus (GEO) database (GSE77661), GEPIA database, and qRT-PCR to screen circRNAs that may be involved in the regulation of PCa progression. Hsa_circ_0030586 was found to be the most significantly upregulated circRNA. Then, we predicted its target genes involved in the PI3K-AKT signaling pathway. We found that hsa_circ_0030586 promotes EMT in PCa via PI3K-AKT signaling, likely by sponging hsa-miR-145-3p.

### Expression of circRNAs in PCa cells

**Figure 1.**
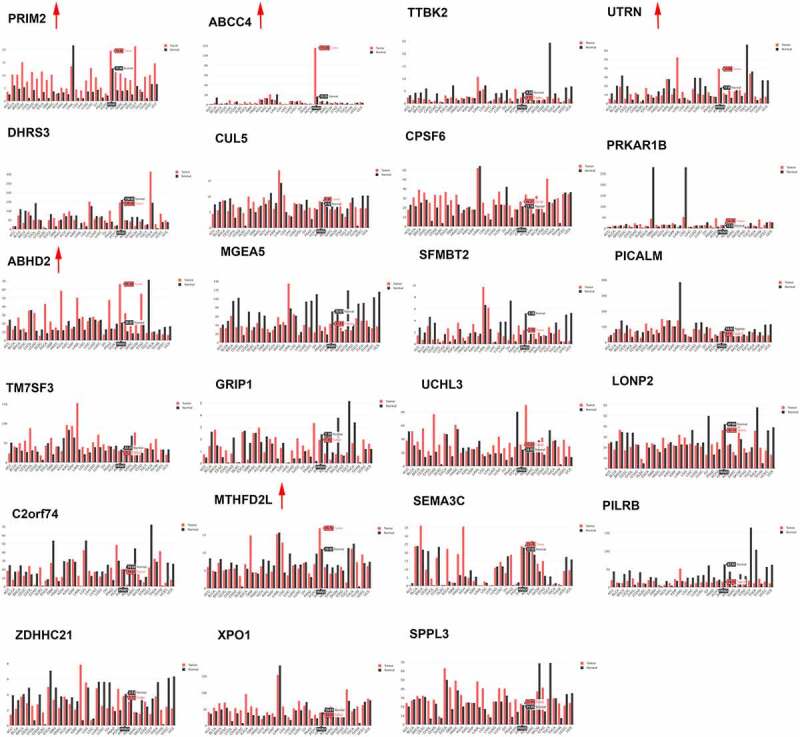
The expression of parent genes of top 24 differentially expressed circRNAs in GEPIA database

**Figure 2.**
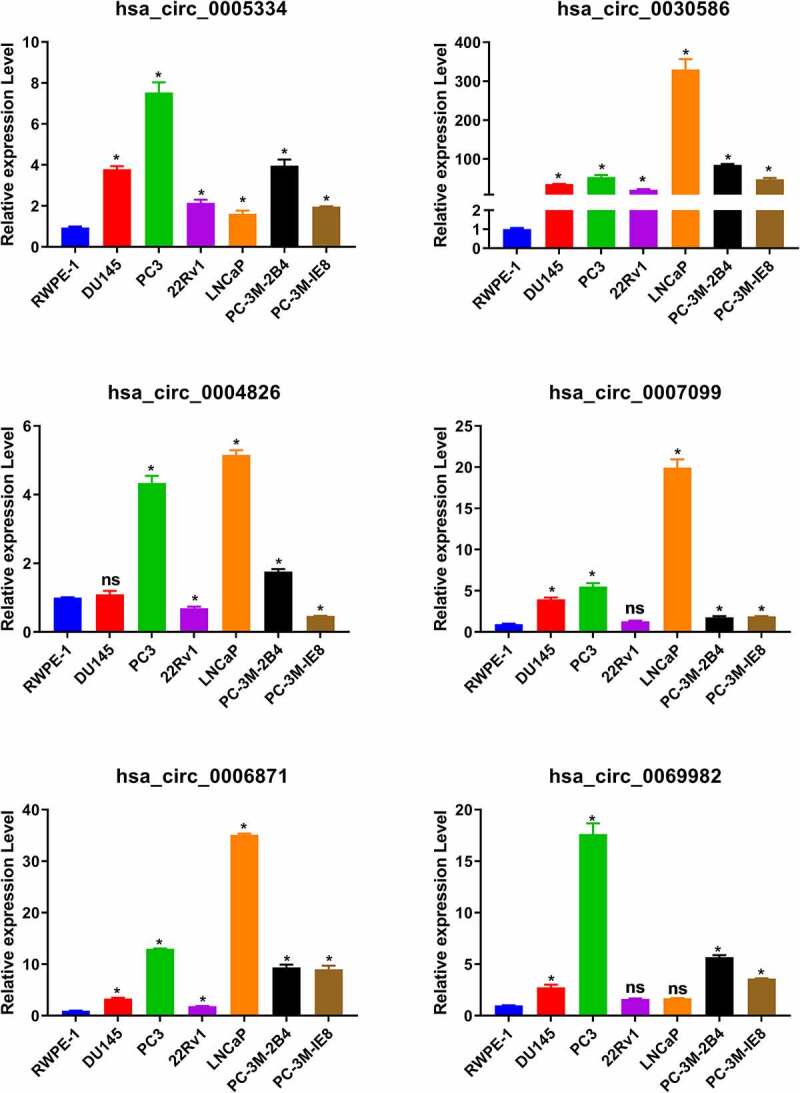
The expression of circRNAs in PCa cells. The expression of hsa_circ_0005334, hsa_circ_0030586, hsa_circ_0004826, hsa_circ_0007099, hsa_circ_0006871, and hsa_circ_0069982 in PCa cells was detected via qRT-PCR. *P < 0.05

By the analysis of the dataset of GSE77661, we got 168 differentially expressed circRNAs between tumor and normal tissues (Table 2). Then we filter the differential expression circRNAs following the criterion: the length of circRNAs is between 300 and 3000 bp; the expression of circRNAs is upregulated in tumor than those in normal tissues. There 53 circRNA fit these criterion, and detail information was listed in Table 3. Furthermore, we searched the expression of parent gene of top 24 circRNAs from Table 3 through GEPIA database. Results showed that the expression of five parent gene (PRIM2, ABCC4, UTRN, ABHD2, and MTHFD2L) was also significant increase in tumor compared to those in normal tissues (Figure 1). The circRNAs corresponding to these parent genes include six circRNAs: hsa_circ_0005334, hsa_circ_0030586, hsa_circ_0004826, hsa_circ_0007099, hsa_circ_0006871, and hsa_circ_0069982). Afterward, qRT-PCR was used to detect these circRNAs expression in normal prostate epithelial cells RWPE-1 and PCa cells. Results indicated that the levels of hsa_circ_0005334, hsa_circ_0030586, and hsa_circ_0006871 were significantly upregulated in all PCa cells compared with those in normal prostate epithelial cells RWPE-1 (all p < 0.05; Figure 2). Among them, hsa_circ_0030586 had more greater difference between PCa cells and RWPE-1 than the others (Figure 2). However, hsa_circ_004826, hsa_circ_0007099, and hsa_circ_0069982 expression in some PCa cells was not significant changes (Figure 2). In addition, the function of hsa_circ_0030586 in PCa had no report, therefore hsa_circ_0030586 was selected for the following studies.

**Table 2.** The differentially expressed circular RNAs

circRNA	circBaseID	geneid	length	PRAD_Normal	PRAD_Tumor	Fold change	Up/Down
chr1:117944807–117984947	hsa_circ_0000119	MAN1A2	648	2.24884	0.197003	0.087602053	Down
chr5:167915606–167921655	hsa_circ_0001550	RARS	534	2.24884	0.197003	0.087602053	Down
chr15:41961025–41962156	hsa_circ_0000591	MGA	1131	1.92758	0.197003	0.102202243	Down
chr15:64791491–64792365	hsa_circ_0000615	ZNF609	874	3.37327	0.394007	0.116802687	Down
chr1:205585605–205593019	hsa_circ_0000175	ELK4	4577	16.7057	1.77303	0.106133236	Down
chr1:207200838–207201024	hsa_circ_0141539	C1orf116	186	0.160632	0.59101	3.679279347	Up
chr1:213341200–213349835	hsa_circ_0005314	RPS6KC1	209	0.321264	1.18202	3.679279347	Up
chr3:125032151–125050082	hsa_circ_0001333	ZNF148	566	1.44569	0.197003	0.136269186	Down
chr5:74109664–74137504	hsa_circ_0007158	FAM169A	673	1.28505	0.197003	0.153303762	Down
chr1:24840803–24841057	hsa_circ_0003553	RCAN3	254	1.44569	0.394007	0.272539064	Down
chr1:23356961–23377013	hsa_circ_0009061	KDM1A	360	1.12442	0.197003	0.175204105	Down
chr1:33760537–33760906	hsa_circ_0009027	ZNF362	238	0.160632	0.394007	2.452854973	Up
chr1:46105881–46108171	hsa_circ_0008774	GPBP1L1	267	0.642527	0.197003	0.306606571	Down
chr1:48821341–48825442	hsa_circ_0008202	SPATA6	285	0.481895	0.197003	0.408808973	Down
chr1:52293467–52299842	hsa_circ_0009076	NRD1	228	0.481895	0.197003	0.408808973	Down
chr1:59805629–59844509	hsa_circ_0006633	FGGY	353	1.12442	0.197003	0.175204105	Down
chr18:19345732–19399607	hsa_circ_0000836	MIB1	1600	1.12442	0.197003	0.175204105	Down
chr19:8520288–8528570	hsa_circ_0006382	HNRNPM	325	1.12442	0.197003	0.175204105	Down
chr1:78177431–78178966	hsa_circ_0012989	USP33	191	0.481895	0.197003	0.408808973	Down
chr4:87967317–87968746	hsa_circ_0001423	AFF1	1021	1.12442	0.197003	0.175204105	Down
chr1:7837219–7838229	hsa_circ_0006354	VAMP3	211	0.642527	0.197003	0.306606571	Down
chr2:206992520–206994966	hsa_circ_0002431	NDUFS1	331	0.963791	0.197003	0.204404274	Down
chr21:47768925–47769734	hsa_circ_0002903	PCNT	312	0.963791	0.197003	0.204404274	Down
chr6:4891946–4892613	hsa_circ_0008285	CDYL	667	0.963791	0.197003	0.204404274	Down
chr8:17601112–17613470	hsa_circ_0083444	MTUS1	2441	0.963791	0.197003	0.204404274	Down
chr10:38092760–38127039	hsa_circ_0093633	ZNF248	29507	0.321264	0.985017	3.06606716	Up
chr8:142264087–142264728	hsa_circ_0001829	TCONS_00015535	641	2.73074	0.59101	0.216428514	Down
chr1:155408117–155429689	hsa_circ_0003247	ASH1L	844	0.803159	0.197003	0.24528518	Down
chr1:33413822–33415375	hsa_circ_0000048	RNF19B	351	0.803159	0.197003	0.24528518	Down
chr10:93841076–93851701	hsa_circ_0004224	CPEB3	297	0.160632	0.394007	2.452854973	Up
chr10:7318853–7327916	hsa_circ_0000211	SFMBT2	434	0.803159	0.197003	0.24528518	Down
chr15:63988322–64008672	hsa_circ_0035796	HERC1	1143	0.803159	0.197003	0.24528518	Down
chr6:16326624–16328701	hsa_circ_0007132	ATXN1	2077	0.803159	0.197003	0.24528518	Down
chr6:3076997–3078169	hsa_circ_0001571	RIPK1	381	0.803159	0.197003	0.24528518	Down
chr11:65267869–65268013	hsa_circ_0096135	MALAT1	144	0.642527	0.197003	0.306606571	Down
chr11:65267990–65268121	hsa_circ_0140958	TCONS_l2_00004578	42	1.60632	0.394007	0.245285497	Down
chr11:65272657–65272846	hsa_circ_0096166	MALAT1	189	1.28505	0.59101	0.459912066	Down
chr13:33091993–33101669	hsa_circ_0000471	N4BP2L2	388	3.21264	0.788014	0.245285497	Down
chr18:12999419–13019205	hsa_circ_0000831	CEP192	1054	1.60632	0.394007	0.245285497	Down
chr11:92085261–92088570	hsa_circ_0000348	FAT3	3309	0.481895	0.197003	0.408808973	Down
chr2:40655612–40657444	hsa_circ_0000994	SLC8A1	1832	4.01579	1.18202	0.294343081	Down
chr12:116668337–116675510	hsa_circ_0000443	MED13L	7173	1.12442	2.36404	2.10245282	Up
chr17:65941524–65944422	hsa_circ_0000798	BPTF	1226	0.642527	0.197003	0.306606571	Down
chr2:227729319–227779067	hsa_circ_0058495	RHBDD1	946	0.642527	0.197003	0.306606571	Down
chr4:146744573–146770713	hsa_circ_0001447	ZNF827	402	0.642527	0.197003	0.306606571	Down
chr4:91229394–91234198	hsa_circ_0127310	FAM190A	1550	0.642527	0.197003	0.306606571	Down
chr5:167915606–167924353	hsa_circ_0001551	RARS	777	0.642527	0.197003	0.306606571	Down
chr12:27149674–27152609	hsa_circ_0007683	TM7SF3	272	0.160632	1.18202	7.358558693	Up
chr12:49525080–49580616	hsa_circ_0000400	TUBA1B	55536	0.321264	1.18202	3.679279347	Up
chr12:56094682–56094938	hsa_circ_0026782	ITGA7	256	1.92758	0.788014	0.40881001	Down
chr9:5968018–5988545	hsa_circ_0138872	KIAA2026	1619	0.642527	0.197003	0.306606571	Down
chr10:32197099–32199491	hsa_circ_0000231	ARHGAP12	794	1.12442	0.394007	0.3504091	Down
chr12:70193988–70195501	hsa_circ_0000419	RAB3IP	242	9.79854	2.75805	0.281475608	Down
chr7:131060182–131084192	hsa_circ_0001746	MKLN1	605	1.12442	0.394007	0.3504091	Down
chrX:79544404–79565732	hsa_circ_0140637	TCONS_l2_00030834	562	1.12442	0.394007	0.3504091	Down
chr1:58971731–59004982	hsa_circ_0002316	OMA1	1381	1.60632	0.59101	0.367927935	Down
chr6:158733082–158735300	hsa_circ_0142313	TCONS_00011401	2218	3.052	1.18202	0.387293578	Down
chr1:78267015–78280916	hsa_circ_0002570	FAM73A	700	0.481895	0.197003	0.408808973	Down
chr1:8601272–8617582	hsa_circ_0002158	RERE	308	0.481895	0.197003	0.408808973	Down
chr10:69785302–69804320	hsa_circ_0007113	HERC4	682	0.481895	0.197003	0.408808973	Down
chr14:20811282–20811436	hsa_circ_0000512	RPPH1	154	0.160632	0.788014	4.905709946	Up
chr14:20811305–20811436	hsa_circ_0000514	RPPH1	131	10.2804	34.0816	3.315201743	Up
chr12:12397195–12397589	hsa_circ_0000378	LRP6	394	0.481895	0.197003	0.408808973	Down
chr12:123983090–123984083	hsa_circ_0007552	RILPL1	341	0.481895	0.197003	0.408808973	Down
chr14:73614502–73614802	hsa_circ_0008521	PSEN1	210	0.321264	0.985017	3.06606716	Up
chr14:75513078–75516421	hsa_circ_0032649	MLH3	3343	0.321264	0.788014	2.452854973	Up
chr13:95768175–95840796	hsa_circ_0030582	ABCC4	1272	0.481895	0.197003	0.408808973	Down
chr17:57430575–57430887	hsa_circ_0005600	YPEL2	312	0.481895	0.197003	0.408808973	Down
chr2:120885263–120932576	hsa_circ_0006834	EPB41 L5	958	0.481895	0.197003	0.408808973	Down
chr22:21288066–21288532	hsa_circ_0001206	CRKL	466	0.481895	0.197003	0.408808973	Down
chr4:39915230–39927553	hsa_circ_0007308	PDS5A	563	0.481895	0.197003	0.408808973	Down
chr5:56160560–56161804	hsa_circ_0001485	MAP3K1	467	0.481895	0.197003	0.408808973	Down
chr7:100410368–100410830	hsa_circ_0001730	EPHB4	362	0.481895	0.197003	0.408808973	Down
chr16:31733946–31734674	hsa_circ_0007059	ZNF720	223	0.963791	0.394007	0.408809586	Down
chr7:91842508–91855996	hsa_circ_0081006	KRIT1	1036	0.481895	0.197003	0.408808973	Down
chr7:91924202–91936970	hsa_circ_0135061	ANKIB1	576	0.481895	0.197003	0.408808973	Down
chr8:141874410–141900868	hsa_circ_0002483	PTK2	482	0.481895	0.197003	0.408808973	Down
chr17:45695715–45696530	hsa_circ_0004622	NPEPPS	264	0.160632	0.394007	2.452854973	Up
chrX:107083899–107097934	hsa_circ_0002153	MID2	812	0.481895	0.197003	0.408808973	Down
chrX:90669902–90673870	hsa_circ_0140700	PABPC5-AS1	303	0.481895	0.197003	0.408808973	Down
chr17:77073511–77073946	hsa_circ_0008114	ENGASE	270	0.160632	0.394007	2.452854973	Up
chr11:33307958–33309057	hsa_circ_0000284	HIPK3	1099	4.33706	1.77303	0.408809193	Down
chr10:112723882–112745523	hsa_circ_0020028	SHOC2	1075	0.963791	0.394007	0.408809586	Down
chr18:29412046–29419420	hsa_circ_0003805	TRAPPC8	236	0.160632	0.394007	2.452854973	Up
chr11:120916382–120930794	hsa_circ_0003302	TBCEL	973	0.963791	0.394007	0.408809586	Down
chr18:42529845–42533305	hsa_circ_0108457	SETBP1	3460	1.12442	0.197003	0.175204105	Down
chr19:17387303–17387718	hsa_circ_0003253	BABAM1	217	1.28505	0.197003	0.153303762	Down
chr19:30476129–30477324	hsa_circ_0000921	C19orf2	215	0.803159	0.394007	0.490571605	Down
chr19:47421744–47425613	hsa_circ_0109744	ARHGAP35	3869	0.160632	0.394007	2.452854973	Up
chr19:47767859–47768203	hsa_circ_0000944	CCDC9	258	0.803159	0.394007	0.490571605	Down
chr19:49416267–49416821	hsa_circ_0002084	NUCB1	277	0.160632	0.394007	2.452854973	Up
chr1:117944807–117957453	hsa_circ_0000117	MAN1A2	472	1.76695	0.788014	0.445974136	Down
chr11:85707868–85742653	hsa_circ_0023923	PICALM	1128	1.28505	0.59101	0.459912066	Down
chr3:171965322–171969331	hsa_circ_0006156	FNDC3B	526	2.89137	1.37902	0.476943456	Down
chr16:68155889–68157024	hsa_circ_0005615	NFATC3	1135	0.803159	0.394007	0.490571605	Down
chr2:203818727–203820481	hsa_circ_0003493	ALS2CR8	215	0.481895	0.197003	0.408808973	Down
chr2:24357988–24369956	hsa_circ_0000982	LOC375190	587	0.803159	0.394007	0.490571605	Down
chr6:57372287–57398317	hsa_circ_0005334	PRIM2	327	0.160632	1.37902	8.584964391	Up
chr13:95813442–95840796	hsa_circ_0030586	ABCC4	1192	0.321264	2.56104	7.971761542	Up
chr2:29006772–29011675	hsa_circ_0007439	PPP1CB	224	0.321264	1.18202	3.679279347	Up
chr15:43120125–43132631	hsa_circ_0008319	TTBK2	320	0.160632	1.18202	7.358558693	Up
chr6:144808683–144814592	hsa_circ_0004826	UTRN	771	0.160632	1.18202	7.358558693	Up
chr2:58449076–58459247	hsa_circ_0001009	FANCL	278	0.160632	0.394007	2.452854973	Up
chr1:12638745–12639440	hsa_circ_0010023	DHRS3	359	0.160632	0.985017	6.132134319	Up
chr11:107916996–107925682	hsa_circ_0024169	CUL5	646	0.160632	0.788014	4.905709946	Up
chr20:30954186–30956926	hsa_circ_0001136	ASXL1	195	0.481895	0.985017	2.044049015	Up
chr20:32207322–32211102	hsa_circ_0003426	CBFA2T2	272	2.40948	0.985017	0.408808955	Down
chr20:57014000–57016139	hsa_circ_0001173	VAPB	258	0.963791	0.394007	0.408809586	Down
chr12:69644908–69656342	hsa_circ_0000417	CPSF6	1599	0.160632	0.788014	4.905709946	Up
chr7:716865–751164	hsa_circ_0008039	PRKAR1B	462	0.160632	0.788014	4.905709946	Up
chr15:89656955–89659752	hsa_circ_0007099	ABHD2	300	0.803159	3.34906	4.169859268	Up
chr10:103552595–103570071	hsa_circ_0019607	MGEA5	1826	0.160632	0.59101	3.679279347	Up
chr22:32874967–32875262	hsa_circ_0008832	FBXO7	295	0.160632	0.985017	6.132134319	Up
chr22:47370185–47393605	hsa_circ_0001252	TBC1D22A	186	0.642527	0.197003	0.306606571	Down
chr10:7285519–7327916	hsa_circ_0017636	SFMBT2	684	0.160632	0.59101	3.679279347	Up
chr11:85707868–85714494	hsa_circ_0023919	PICALM	451	0.160632	0.59101	3.679279347	Up
chr12:27132716–27143560	hsa_circ_0025705	TM7SF3	499	0.160632	0.59101	3.679279347	Up
chr3:182788786–182789145	hsa_circ_0008550	MCCC1	270	0.160632	0.59101	3.679279347	Up
chr12:66909398–66935730	hsa_circ_0027450	GRIP1	588	0.160632	0.59101	3.679279347	Up
chr13:76134888–76143643	hsa_circ_0000494	UCHL3	420	0.160632	0.59101	3.679279347	Up
chr13:95829960–95840796	hsa_circ_0006871	ABCC4	464	0.160632	0.59101	3.679279347	Up
chr16:48311248–48337216	hsa_circ_0008558	LONP2	554	0.160632	0.59101	3.679279347	Up
chr2:61389976–61391675	hsa_circ_0005338	C2orf74	482	0.160632	0.59101	3.679279347	Up
chr4:102117072–102117273	hsa_circ_0124877	PPP3CA	201	0.160632	0.394007	2.452854973	Up
chr4:75040222–75091111	hsa_circ_0069982	MTHFD2L	662	0.160632	0.59101	3.679279347	Up
chr7:80418621–80440017	hsa_circ_0004365	SEMA3C	907	0.321264	1.18202	3.679279347	Up
chr7:99952765–99953427	hsa_circ_0005925	PILRB	662	0.160632	0.59101	3.679279347	Up
chr9:14639893–14680160	hsa_circ_0008952	ZDHHC21	796	0.160632	0.59101	3.679279347	Up
chr2:61749745–61761038	hsa_circ_0001017	XPO1	307	0.481895	1.57603	3.270484234	Up
chr12:121220457–121222396	hsa_circ_0003472	SPPL3	312	0.321264	0.985017	3.06606716	Up
chr13:95813442–95822882	hsa_circ_0006659	ABCC4	728	0.803159	2.16704	2.698145697	Up
chr14:21971315–21972024	hsa_circ_0000523	METTL3	623	0.481895	1.18202	2.452857988	Up
chr1:52981562–52992045	hsa_circ_0012553	ZCCHC11	975	0.160632	0.394007	2.452854973	Up
chr5:72370568–72373320	hsa_circ_0002490	FCHO2	268	2.57011	0.59101	0.229955138	Down
chr11:93480462–93493024	hsa_circ_0096789	C11orf54	871	0.160632	0.394007	2.452854973	Up
chr12:1887019–1893239	hsa_circ_0025006	ADIPOR2	741	0.160632	0.394007	2.452854973	Up
chr13:95686858–95735544	hsa_circ_0030569	ABCC4	1335	0.160632	0.394007	2.452854973	Up
chr14:38256672–38266152	hsa_circ_0101775	TTC6	763	0.160632	0.394007	2.452854973	Up
chr14:96986391–96991728	hsa_circ_0002120	PAPOLA	323	0.160632	0.394007	2.452854973	Up
chr15:80390757–80415142	hsa_circ_0000642	ZFAND6	544	0.160632	0.394007	2.452854973	Up
chr16:48290520–48311390	hsa_circ_0105461	LONP2	915	0.160632	0.394007	2.452854973	Up
chr18:33606862–33613800	hsa_circ_0000842	RPRD1A	638	0.160632	0.394007	2.452854973	Up
chr2:100623093–100628033	hsa_circ_0055855	AFF3	895	0.160632	0.394007	2.452854973	Up
chr2:153000340–153006743	hsa_circ_0117652	STAM2	664	0.160632	0.394007	2.452854973	Up
chr2:36623756–36669878	hsa_circ_0002346	CRIM1	538	0.160632	0.394007	2.452854973	Up
chr21:30698379–30702014	hsa_circ_0061395	BACH1	1542	0.160632	0.394007	2.452854973	Up
chr7:27668989–27672064	hsa_circ_0003958	HIBADH	232	0.481895	0.197003	0.408808973	Down
chr3:119222378–119236162	hsa_circ_0001330	TIMMDC1	347	0.160632	0.394007	2.452854973	Up
chr3:18419661–18462483	hsa_circ_0064555	SATB1	1599	0.160632	0.394007	2.452854973	Up
chr7:90376995–90419967	hsa_circ_0001722	CDK14	175	0.160632	0.394007	2.452854973	Up
chr3:56626997–56628056	hsa_circ_0001313	CCDC66	468	0.160632	0.394007	2.452854973	Up
chr3:71090478–71102924	hsa_circ_0008234	FOXP1	587	0.160632	0.394007	2.452854973	Up
chr3:93714717–93722752	hsa_circ_0006135	ARL13B	321	0.160632	0.394007	2.452854973	Up
chr8:131370262–131374017	hsa_circ_0085616	ASAP1	229	0.160632	0.394007	2.452854973	Up
chr4:10099334–10105610	hsa_circ_0003550	WDR1	420	0.160632	0.394007	2.452854973	Up
chr5:127474288–127488497	hsa_circ_0006034	SLC12A2	955	0.160632	0.394007	2.452854973	Up
chr6:42618021–42620383	hsa_circ_0076387	UBR2	307	0.160632	0.394007	2.452854973	Up
chr9:114296018–114296633	hsa_circ_0001962	ZNF483	220	0.642527	0.197003	0.306606571	Down
chr9:134381500–134381840	hsa_circ_0001897	POMT1	158	0.160632	0.59101	3.679279347	Up
chr6:87925620–87928449	hsa_circ_0004058	ZNF292	370	0.321264	0.788014	2.452854973	Up
chr9:19286766–19305525	hsa_circ_0005684	DENND4C	4794	0.803159	0.197003	0.24528518	Down
chr9:96233422–96261168	hsa_circ_0001875	FAM120A	556	0.321264	0.788014	2.452854973	Up
chr9:79244107–79251473	hsa_circ_0087236	PRUNE2	186	0.481895	0.197003	0.408808973	Down
chr9:88918000–88919862	hsa_circ_0007037	ZCCHC6	208	0.160632	0.394007	2.452854973	Up
chr11:36248634–36248980	hsa_circ_0006988	LDLRAD3	346	0.803159	1.97003	2.452851801	Up
chr21:37619814–37620866	hsa_circ_0001187	DOPEY2	301	0.481895	0.985017	2.044049015	Up
chr6:30618770–30619243	hsa_circ_0006109	None	473	0.481895	0.985017	2.044049015	Up
chr1:247319707–247323115	hsa_circ_0112879	ZNF124	1186	0.963791	1.97003	2.044042744	Up

**Table 3.** The upregulated differentially expressed circular RNAs (limite the length between 300 and 3000 bp)

circRNA	circBaseID	geneid	length	PRAD_Normal	PRAD_Tumor	Fold change	Up/Down
chr6:57372287–57398317	hsa_circ_0005334	PRIM2	327	0.160632	1.37902	8.584964391	Up
chr13:95813442–95840796	hsa_circ_0030586	ABCC4	1192	0.321264	2.56104	7.971761542	Up
chr15:43120125–43132631	hsa_circ_0008319	TTBK2	320	0.160632	1.18202	7.358558693	Up
chr6:144808683–144814592	hsa_circ_0004826	UTRN	771	0.160632	1.18202	7.358558693	Up
chr1:12638745–12639440	hsa_circ_0010023	DHRS3	359	0.160632	0.98502	6.132134319	Up
chr11:107916996–107925682	hsa_circ_0024169	CUL5	646	0.160632	0.78801	4.905709946	Up
chr12:69644908–69656342	hsa_circ_0000417	CPSF6	1599	0.160632	0.78801	4.905709946	Up
chr7:716865–751164	hsa_circ_0008039	PRKAR1B	462	0.160632	0.78801	4.905709946	Up
chr15:89656955–89659752	hsa_circ_0007099	ABHD2	300	0.803159	3.34906	4.169859268	Up
chr10:103552595–103570071	hsa_circ_0019607	MGEA5	1826	0.160632	0.59101	3.679279347	Up
chr10:7285519–7327916	hsa_circ_0017636	SFMBT2	684	0.160632	0.59101	3.679279347	Up
chr11:85707868–85714494	hsa_circ_0023919	PICALM	451	0.160632	0.59101	3.679279347	Up
chr12:27132716–27143560	hsa_circ_0025705	TM7SF3	499	0.160632	0.59101	3.679279347	Up
chr12:66909398–66935730	hsa_circ_0027450	GRIP1	588	0.160632	0.59101	3.679279347	Up
chr13:76134888–76143643	hsa_circ_0000494	UCHL3	420	0.160632	0.59101	3.679279347	Up
chr13:95829960–95840796	hsa_circ_0006871	ABCC4	464	0.160632	0.59101	3.679279347	Up
chr16:48311248–48337216	hsa_circ_0008558	LONP2	554	0.160632	0.59101	3.679279347	Up
chr2:61389976–61391675	hsa_circ_0005338	C2orf74	482	0.160632	0.59101	3.679279347	Up
chr4:75040222–75091111	hsa_circ_0069982	MTHFD2L	662	0.160632	0.59101	3.679279347	Up
chr7:80418621–80440017	hsa_circ_0004365	SEMA3C	907	0.321264	1.18202	3.679279347	Up
chr7:99952765–99953427	hsa_circ_0005925	PILRB	662	0.160632	0.59101	3.679279347	Up
chr9:14639893–14680160	hsa_circ_0008952	ZDHHC21	796	0.160632	0.59101	3.679279347	Up
chr2:61749745–61761038	hsa_circ_0001017	XPO1	307	0.481895	1.57603	3.270484234	Up
chr12:121220457–121222396	hsa_circ_0003472	SPPL3	312	0.321264	0.98502	3.06606716	Up
chr13:95813442–95822882	hsa_circ_0006659	ABCC4	728	0.803159	2.16704	2.698145697	Up
chr14:21971315–21972024	hsa_circ_0000523	METTL3	623	0.481895	1.18202	2.452857988	Up
chr1:52981562–52992045	hsa_circ_0012553	ZCCHC11	975	0.160632	0.39401	2.452854973	Up
chr11:93480462–93493024	hsa_circ_0096789	C11orf54	871	0.160632	0.39401	2.452854973	Up
chr12:1887019–1893239	hsa_circ_0025006	ADIPOR2	741	0.160632	0.39401	2.452854973	Up
chr13:95686858–95735544	hsa_circ_0030569	ABCC4	1335	0.160632	0.39401	2.452854973	Up
chr14:38256672–38266152	hsa_circ_0101775	TTC6	763	0.160632	0.39401	2.452854973	Up
chr14:96986391–96991728	hsa_circ_0002120	PAPOLA	323	0.160632	0.39401	2.452854973	Up
chr15:80390757–80415142	hsa_circ_0000642	ZFAND6	544	0.160632	0.39401	2.452854973	Up
chr16:48290520–48311390	hsa_circ_0105461	LONP2	915	0.160632	0.39401	2.452854973	Up
chr18:33606862–33613800	hsa_circ_0000842	RPRD1A	638	0.160632	0.39401	2.452854973	Up
chr2:100623093–100628033	hsa_circ_0055855	AFF3	895	0.160632	0.39401	2.452854973	Up
chr2:153000340–153006743	hsa_circ_0117652	STAM2	664	0.160632	0.39401	2.452854973	Up
chr2:36623756–36669878	hsa_circ_0002346	CRIM1	538	0.160632	0.39401	2.452854973	Up
chr21:30698379–30702014	hsa_circ_0061395	BACH1	1542	0.160632	0.39401	2.452854973	Up
chr3:119222378–119236162	hsa_circ_0001330	TIMMDC1	347	0.160632	0.39401	2.452854973	Up
chr3:18419661–18462483	hsa_circ_0064555	SATB1	1599	0.160632	0.39401	2.452854973	Up
chr3:56626997–56628056	hsa_circ_0001313	CCDC66	468	0.160632	0.39401	2.452854973	Up
chr3:71090478–71102924	hsa_circ_0008234	FOXP1	587	0.160632	0.39401	2.452854973	Up
chr3:93714717–93722752	hsa_circ_0006135	ARL13B	321	0.160632	0.39401	2.452854973	Up
chr4:10099334–10105610	hsa_circ_0003550	WDR1	420	0.160632	0.39401	2.452854973	Up
chr5:127474288–127488497	hsa_circ_0006034	SLC12A2	955	0.160632	0.39401	2.452854973	Up
chr6:42618021–42620383	hsa_circ_0076387	UBR2	307	0.160632	0.39401	2.452854973	Up
chr6:87925620–87928449	hsa_circ_0004058	ZNF292	370	0.321264	0.78801	2.452854973	Up
chr9:96233422–96261168	hsa_circ_0001875	FAM120A	556	0.321264	0.78801	2.452854973	Up
chr11:36248634–36248980	hsa_circ_0006988	LDLRAD3	346	0.803159	1.97003	2.452851801	Up
chr21:37619814–37620866	hsa_circ_0001187	DOPEY2	301	0.481895	0.98502	2.044049015	Up
chr6:30618770–30619243	hsa_circ_0006109	None	473	0.481895	0.98502	2.044049015	Up
chr1:247319707–247323115	hsa_circ_0112879	ZNF124	1186	0.963791	1.97003	2.044042744	Up

### Validation of the circular structure of hsa_circ_0030586

**Figure 3.**
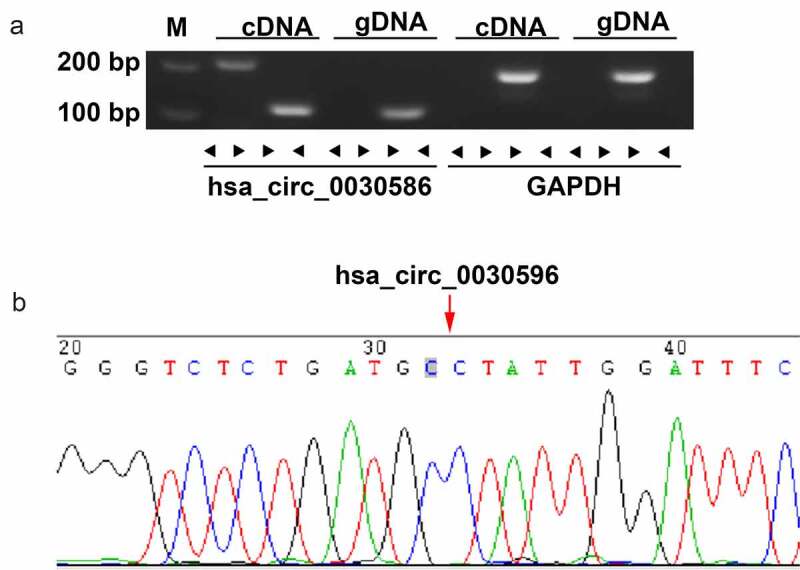
Validation of the circular structure of hsa_circ_0030586. (a) Confirmation of the circular structure of hsa_circ_0030586 in PCa cells. RT-PCR was used to detect circular and linear cDNA using divergent and convergent primers, respectively. Primers targeting GAPDH and genomic DNA were considered as negative controls. (b) The junction site of hsa_circ_0030586 was confirmed via Sanger sequencing

The circular structure of hsa_circ_0030586 was confirmed via agarose gel electrophoresis and Sanger sequencing. The results showed that hsa_circ_0030586 can only be amplified in cDNA by adopting divergent primers but not genomic DNA (Figure 3a). The sequence of the splice junction was further confirmed via Sanger sequencing (Figure 3b). These results convincingly showed that hsa_circ_0030586 can correctly form a ring structure.

### Interfering of hsa_circ_0030586 suppressed the proliferation, migration, and invasion of PCa cells

**Figure 4.**
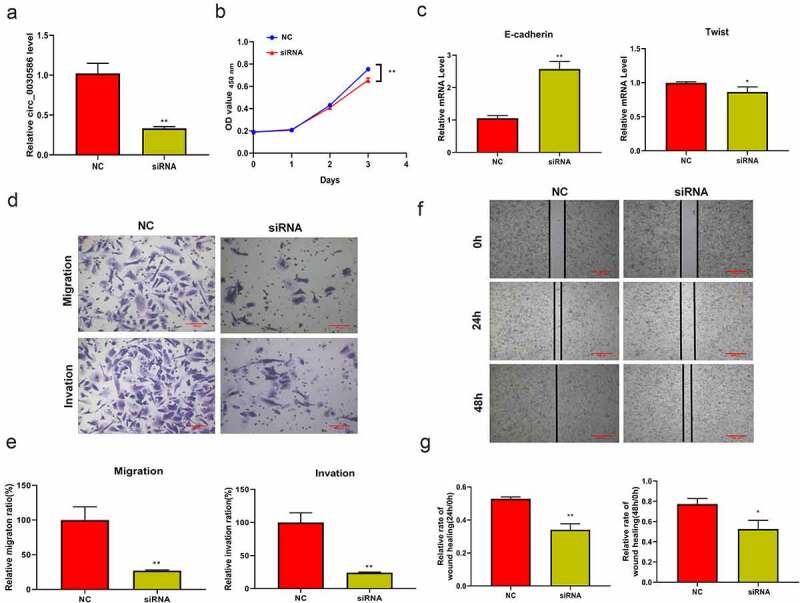
Interfering with hsa_circ_0030586 suppressed the proliferation, migration, and invasion of PCa cells. (a) Transient interference fragments significantly reduced the expression of hsa_circ_0030586. (b) Transfection with hsa_circ_0030586 siRNA significantly inhibits cell viability at 72 h. (c) The EMT-related gene E-cadherin was significantly upregulated, and Twist was significantly downregulated in cells transfected with hsa_circ_0030586 siRNA. (d–e) Transfection with hsa_circ_0030586 siRNA significantly inhibited the migration and invasion of PC3 cells (scare bar: 100 μm). (f–g) Transfection with hsa_circ_0030586 siRNA significantly inhibited the wound healing capacity of PC3 cells (scare bar: 500 μm). *P < 0.05; ** P < 0.01

qRT-PCR results showed that transient interference fragments designed for hsa_circ_0030586 can significantly reduce its expression (p < 0.01; Figure 4a). Furthermore, the CCK8 assay found that the inhibition of hsa_circ_0030586 expression can significantly inhibit cell viability at 72 h (p < 0.01; Figure 4b). Moreover, qRT-PCR results showed that the EMT-related gene E-cadherin was significantly upregulated in the cells transfected with hsa_circ_0030586 siRNA compared to those transfected with siRNA NC (p < 0.01; Figure 4c), whereas Twist was significantly downregulated (p < 0.05; Figure 4c). The transwell assay and the wound healing assay showed that transfection with hsa_circ_0030586 siRNA can effectively inhibit migration (p < 0.01), invasion (p < 0.01), and the wound healing capacity (p < 0.05) of PC3 cells (Figure 4d–4g)

### mRNA sequencing analysis

**Figure 5.**
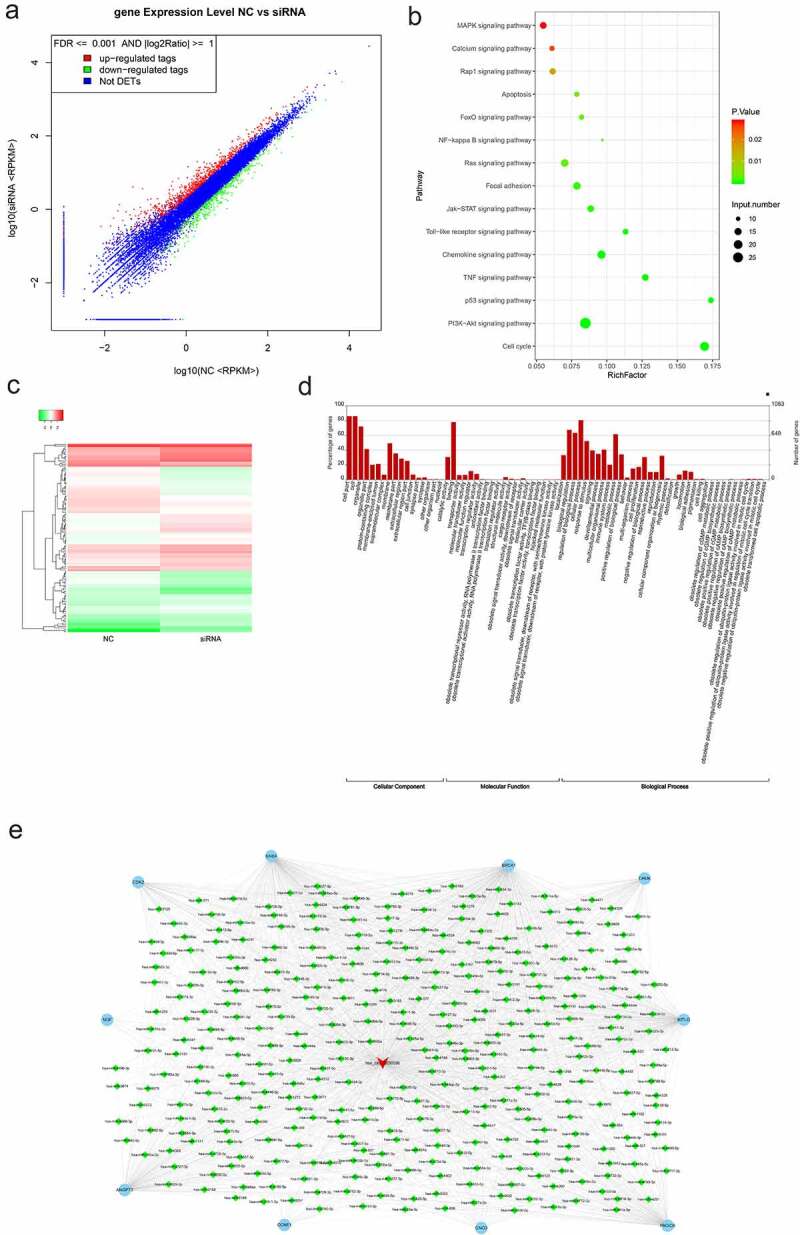
mRNA sequencing analysis. (a) The scatter plot showed 1,202 differentially expressed mRNAs between the NC and siRNA groups, 792 were upregulated, and 410 were downregulated. (b) KEGG analysis showed that differentially expressed mRNAs are mainly enriched in PI3K-AKT, cell cycle, p53, and other signaling pathways associated with tumor cell proliferation, apoptosis, and metastasis. (c) Heat map showing the differentially expressed mRNAs between the NC and siRNA groups. (d) GO analysis showed the distribution of these differentially expressed genes on the BP (biological process), CC (cellular component), and MF (molecular function) rich GO Terms. (e) The circRNAs–miRNAs–mRNAs competing endogenous RNA (ceRNA) networks involved in the PI3K-AKT signaling pathway

The scatter plot (Figure 5a) showed 1,202 differentially expressed mRNAs between the NC and siRNA groups, among which 792 were upregulated and 410 were downregulated. KEGG analysis found that these differentially expressed mRNAs were mainly enriched in PI3K-AKT, cell cycle, p53 signaling, and other signaling pathways associated with tumor cell proliferation, apoptosis, and metastasis (Figure 5b). A heat map was drawn with 114 differentially expressed mRNAs involved in the 15 signaling pathways identified via KEGG analysis (Figure 5c). GO analysis showed that differentially expressed mRNAs were significantly enriched in various biological processes, including cellular processes, biological regulation, and binding (Figure 5d). Furthermore, based on the competing endogenous RNA (ceRNA) mechanism, the interactive networks were drawn with the miRNAs and target genes involved in the PI3K-AKT signaling pathway and their expression was consistent with that of hsa_circ_0030586 (Figure 5e).

### Hsa_circ_0030586 activates the PI3K-AKT signaling pathway and promotes EMT

**Figure 6.**
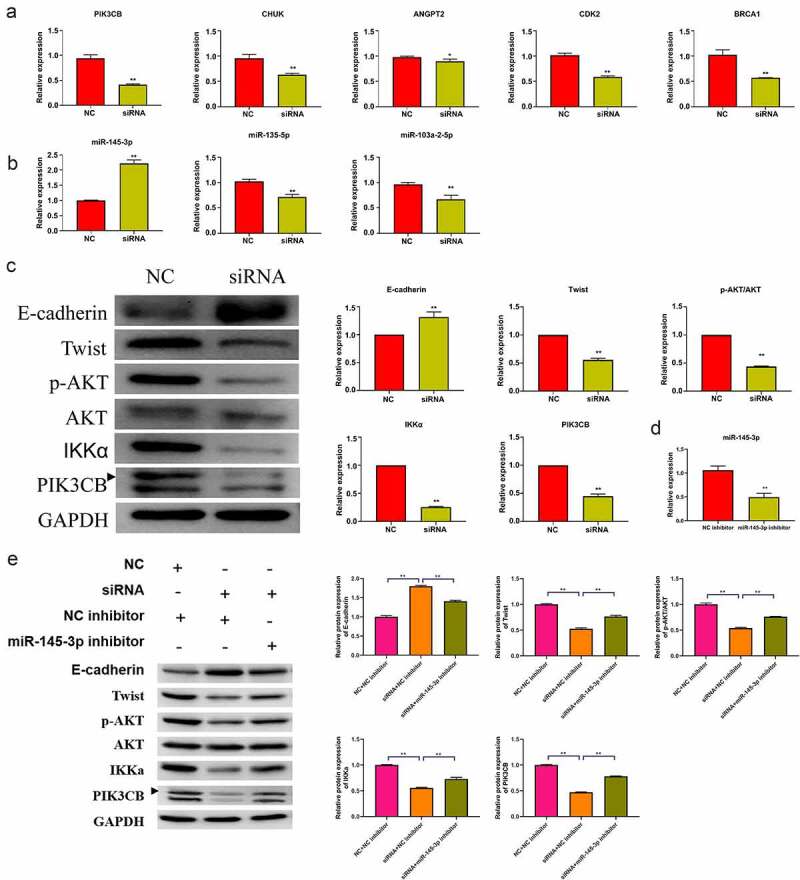
Hsa_circ_0030586 activates the PI3K-AKT signaling and promotes EMT. (a) The relative expression of five key molecules involved in the PI3K-AKT signaling pathway was detected by qRT-PCR. (b) The relative expression of three miRNAs targeting PIK3CB was detected by qRT-PCR. (c) The expression of E-cadherin, p-AKT, AKT, IKKα, PIK3CB, and Twist was tested via Western blot. GAPDH was used as an internal standard. (d) miR-145-3p expression was dectected in NC inhibitor and miR-145-3p inhibitor group; (e) The protein expression of E-cadherin, Twist, p-AKT, AKT, IKKα, and PIK3CB was tested via Western blot. GAPDH was used as an internal standard. *P < 0.05; ** P < 0.01

Five key molecules involved in the PI3K-AKT signaling pathway in the network were selected for qRT-PCR verification. The relative expressions of ANGPT2, CHUK, PIK3CB, CDK2, and BRCA1 were significantly downregulated in the hsa_circ_0030586 siRNA group compared to siRNA NC group (all p < 0.05; Figure 6a). Among them, PIK3CB showed the highest downregulation folds, and it was an important upstream regulatory molecule of the PI3K-AKT signaling pathway (p < 0.01; Figure 6a). Furthermore, the qRT-PCR verification of miRNA targeting PIK3CB was conducted. The miRNAs that were the binding sites of circRNA and miRNA > 1 that had been reported as having tumor suppressor effects in PCa [17,19–22] and were significantly negatively correlated with PIK3CB predicted by ENCORI (http://starbase.sysu.edu.cn/panMirCoExp.php#) were selected. Hsa-miR-145-3p, hsa-miR-135a-5p, and hsa-miR-103a-2-5p were screened out and verified. The results showed that the relative expression of hsa-miR-145-3p was significantly upregulated in the hsa_circ_0030586 siRNA group compared to siRNA NC group (p < 0.01), which was in line with our expectations (Figure 6b). Contrary to expectations, the relative expressions of hsa-miR-135a-5p and hsa-miR-103a-2-5p were significantly downregulated (p < 0.01; Figure 6b). Meanwhile, a bioinformatics analysis conducted using miRanda version 3.3a also found that hsa-miR-145-3p can target and regulate BRCA1 and CDK2, which demonstrated that hsa-miR-145-3p played an important role in the process of hsa_circ_0030586, activating the PI3K-AKT signaling pathway. After interfering with hsa_circ_0030586, the expression of p-AKT, AKT, IKKα, and PIK3CB in the PI3K-AKT signaling pathway and the expression of the EMT markers E-cadherin and Twist were measured by WB. The relative protein expression of p-AKT/AKT, IKKα, PIK3CB, and Twist was significantly downregulated, whereas that of E-cadherin was significantly upregulated in siRNA group compared to those in siRNA NC group (all p < 0.01; Figure 6c). The results indicated that interference with hsa_circ_0030586 can inhibit the PI3K-AKT signaling pathway and EMT in PCa cells. Furthermore, PC3 cells were transferred with an NC inhibitor and miR-145-3p inhibitor fragments for 48 h. The qRT-PCR results showed that miR-145-3p was significantly downregulated after transfection with miR-145-3p inhibitor than those in NC inhibitor group (p < 0.01; Figure 6d). Moreover, the hsa_circ_0030586 interference fragment was transfected alone or combined with a miR-145-3p inhibitor to transfect into PC3 cells for 48 h. Transfecting an hsa_circ_0030586 interference fragment alone could downregulate the expression of p-AKT/AKT, IKKα, PIK3CB, and Twist and upregulate the expression of E-cadherin compared to the NC + NC inhibitor group (p < 0.01; Figure 6e). It was found that the hsa_circ_0030586 interference fragment combined with miR-145-3p inhibitor transfection could reverse the effects caused by hsa_circ_003058 interfering (p < 0.01; Figure 6e).

### The tumor formation test in nude mice confirmed that hsa_circ_0030586 activates the PI3K-AKT signaling pathway and promotes EMT

**Figure 7.**
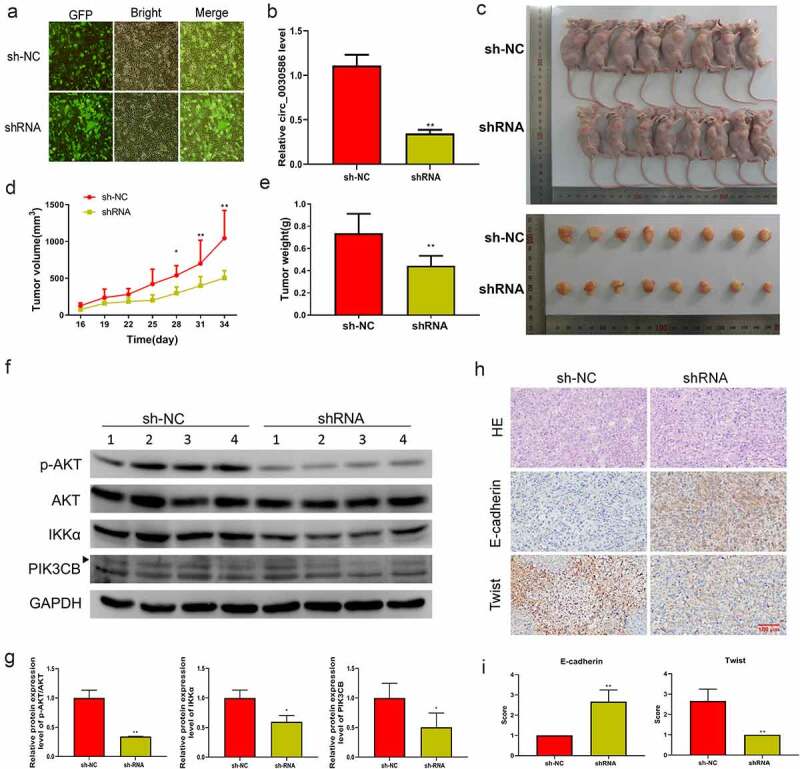
Interference with hsa_circ_0030586 inhibited tumorigenesis in nude mice. (a) Immunofluorescence staining shows the fluorescence rate of the cells. (b) Hsa_circ_0030586 expression in the shRNA and shNC groups was detected by qRT-PCR. (c) Nude mice and tumors injected with PC3-shRNA cells or PC3-shNC cells. (d, e) Tumor volumes (d) and weights (e) were calculated after nude mice were injected with PC3-shRNA cells or PC3-shNC cells. (f, g) p-AKT, AKT, IKKα, and PIK3CB protein expression was detected via Western blot. (h) HE and immunohistochemistry were used to detect the tumor morphology and expression of E-cadherin and Twist (scare bar: 100 μm). *p < 0.05; **p < 0.01

Male BALB/c nude mice were injected with PC3-shNC (shNC group) or PC3-sh (shRNA group) to induce tumorigenesis. A stable lentiviral cell line that interfered with hsa_circ_0030586 was constructed, and immunofluorescence staining showed that the fluorescence rate of the cells reached over 90% (Figure 7a). Furthermore, qRT-PCR results confirmed that the relative expression of hsa_circ_0030586 in the shRNA group was significantly downregulated compared with that in the shNC group (p < 0.01; Figure 7b). The tumor volume and the body weight of mice in the shRNA group were significantly reduced compared with those in the shNC group (all p < 0.05; Figure 6c–6e). WB analysis showed that, after interfering with hsa_circ_0030586, the protein expression levels of p-AKT/AKT, IKKα, and PIK3CB were significantly decreased (all p < 0.05; figure 6f–6g), indicating that the PI3K-AKT signaling pathway was significantly inhibited. The morphology of the tumor in two groups was observed via hematoxylin and eosin staining, which showed that the cancer cells of each group were of different sizes and shapes and arranged in nests, the nuclei were mostly oval, and there were necrotic areas of different sizes in the center of the cancer nests (Figure 6h). Moreover, IHC showed that E-cadherin expression was significantly upregulated and the expression of Twist was significantly downregulated after interfering with hsa_circ_0030586 (Figure 6h–6i), further indicating that interference with hsa_circ_0030586 inhibited tumor EMT.

## Discussion

Despite improvements in diagnostic and therapeutic treatments, several patients still developed advanced PCa at the time of diagnosis and missed the chance for in-time treatment because of a lack of effective early diagnostic markers [23,24]. Therefore, it is necessary to explore new early diagnostic molecular markers for PCa [25,26].

CircRNAs are potent regulators of various diseases. Recently, several circRNAs have been reported to regulate PCa development, mainly by acting as miRNA sponges and regulating target gene expression. Chen et al. identified 1,233 unique fusion genes and 76,311 distinct circRNAs from 144 localized prostate tumors via ultra-deep non-poly-A RNA-seq. In the differentially expressed circRNAs, the global burden of circRNAs was related to tumor aggressivity and 171 circRNAs were essential to PCa cell proliferation [27]. Circ_0044516 was reported to play an important role in PCa cell survival and metastasis and may become a potential biomarker of PCa [28]. It was reported that hsa_circ_0004870 is involved in the progression of enzalutamide resistance in CRPC [29]. Feng et al. verified that circ0005276 interacted with FUS binding protein to activate the transcription of XIAP and promote proliferation and migration in PCa cells [30]. circ_KATNAL1 was reported to play an anticancer role by regulating the miR-145-3p/WISP1 axis in PCa cells and may be the target for PCa diagnosis and treatment [31]. These studies suggested that circRNAs play an important role in PCa development. Although plenty of circRNAs have been identified in PCa, some of them have not been thoroughly investigated yet, and their mechanism remains to be further studied.

In this study, hsa_circ_0030586 was significantly upregulated in PCa cells, and interfering with it suppressed the proliferation, migration, and invasion of PC3 cells. The in vivo study also proved that interfering with hsa_circ_0030586 significantly suppressed PCa cell growth. Our study suggested that hsa_circ_0030586 could promote cancer cell progression in PCa, which is consistent with previous studies arguing that circRNAs promote PCa progression [32–35]. By contrast, circCRKL was lowly expressed in PCa tissues, and there was an upregulated expression of KLF5 by sponging miR-141 to inhibit PCa progression [36]. Another study reported that circUCK2 expression was downregulated in enzalutamide-resistant (EnzR) cells, and circUCK2 overexpression inhibited the EnzR cell growth [37]. This evidence suggested that circRNAs are abnormally expressed in PCa, functioning through various mechanisms.

CircRNAs act as miRNA sponges to control their expression and affect downstream target gene function [38,39]. For example, CircHIPK3 acts as a miR-338-3p sponge and promotes the expression of Cdc25B and Cdc2 to increase the proliferation ability of PCa [40]. CircFMN2 acts as a miR-1238 sponge to promote LIM-homeobox Gene 2 expression, thereby regulating PCa progression [41]. In this study, through a ceRNA mechanism, five key molecules involved in the PI3K-AKT signaling pathway were selected for qRT-PCR validation. We found that the relative expressions of ANGPT2, CHUK, PIK3CB, CDK2, and BRCA1 were significantly downregulated after interfering with hsa_circ_0030586. Among them, PIK3CB showed the highest downregulation, and it is an important upstream regulatory molecule of the PI3K-AKT signaling pathway. The PIK3CB gene was found to be involved in the progress of PCa in cell culture and nude mouse xenograft models [42]. Additionally, elevated PI3K activity is one of the major mechanisms for many types of human cancers, including PCa [43,44]. It has been reported that the long noncoding DANCR promotes PCa via the FAK/PI3K/AKT/GSK3β/snail axis by targeting miR-185-5p [45]. Another study revealed that lncRNA-SNHG1 regulates Wnt/β-catenin and the PI3K/AKT/mTOR signaling axis to affect proliferation, apoptosis, and autophagy in PCa cells [46]. These studies supported our speculation that hsa_circ_0030586 may be regulating PCa progression via PI3K/AKT signaling. Furthermore, the qRT-PCR verification of miRNA targeting PIK3CB was conducted, and the relative expression of hsa-miR-145-3p was significantly upregulated after interfering with hsa_circ_0030586, which was in agreement with a ceRNA mechanism. Furthermore, the hsa_circ_003058 interference fragment combined with miR-145-3p. The inhibition of transfection could reverse the effects caused by hsa_circ_003058 interference, which demonstrated that the effects of hsa_circ_003058 on the PI3K-AKT pathway and EMT are dependent on miR-145-3p. The role of miR-145-3p in PCa has been largely reported. For example, miR-145-3p was found to be closely related to PCa and to be a target of metadherin (MTDH) [19], miR-145-3p may regulate the expression of hsa_circRNA_008068 and hsa_circRNA_406557 in LNCaP cells [17], and miR-145-5p and miR-145-3p were downregulated in CRPC [20]. For further validation, the expression of key proteins p-AKT, AKT, IKKα, and PIK3CB in the PI3K-AKT signaling pathway and the expression of EMT markers E-cadherin and Twist were detected by WB. It was found that the relative protein expression of p-AKT/AKT, IKKα, PIK3CB, and Twist was significantly downregulated, whereas E-cadherin was significantly upregulated after interfering with hsa_circ_0030586. The above results were verified by both in vitro and in vivo studies. These results indicated that interfering with hsa_circ_0030586 may inhibit EMT via PI3K-AKT signaling in PCa cells.

However, studies on clinical tissue specimens were not reported in this article. In further studies, we will conduct an in-depth investigation of hsa_circ_0030586 using clinical specimens. Additionally, more studies are needed to explore the function and potential mechanism of downregulated circRNAs in GSE77661.

## Conclusions

In conclusion, hsa_circ_0030586 is highly expressed in PCa cells and may sponge miR-145-3p to promote EMT via PI3K-AKT signaling. It is suggested that hsa_circ_0030586 can be used as a molecular marker for PCa, helping its early diagnosis and treatment.
